# A case series analysing patients with dental anxiety: a patient-centered model based on psychological profiling

**DOI:** 10.12688/f1000research.20712.2

**Published:** 2020-09-04

**Authors:** Riccardo Tizzoni, Laura Veneroni, Alfonso D'Aloia, Marta Tizzoni, Carlo Alfredo Clerici

**Affiliations:** 1 Independent Researcher, Milano, Italy; 2 Paediatric Oncology Unit, Fondazione IRCCS Istituto Nazionale Tumori, Milano, Italy; 3 Department of Anesthesiology, Intensive Care and Pain Therapy Dept, ASST G. Pini-CTO Hospital, Milano, Italy; 4 Department of Oncology and Haematology-Oncology, University of Milano, Milano, Italy; 5 Clinical Psychology Unit, Fondazione IRCCS Istituto Nazionale Tumori, Milano, Italy

**Keywords:** dental anxiety, sedation, analgesia, compliance, patient-centered model

## Abstract

Anxiety and distress can jeopardize dental care experience of patients and may affect the clinical result. Although a wide range of sedation and analgesia techniques are currently available to relieve distress and pain during dental procedures, operative models to choose the most effective sedation-analgesic strategies are still insufficient.

This case series proposes a patient-centered model to optimize patients’ cooperation during dental care delivery. We describe how to achieve correct anaesthesia by using the least sedative procedure, accounting for the dental procedure needed and patient’s psychological profile.

Five patients were considered as paradigmatic to show the balance between patients’ subjective experiences and the clinical procedures: a patient with low stress, good compliance (case 1); a patient with moderate stress and reduction in compliance (case 2); anxious patient (case 3); a patient with acute anxiety and emotional distress (case 4); anguished patient (case 5).

A multimodal treatment of emotional and behavioural condition and a patient-centered model approach contributed to achieve the best patient satisfaction in the five cases detailed here.

## Introduction

An increasing number of patients are undergoing day-case dental procedures or surgeries, and in some cases they may experience significant emotional upset from either consultation or dental therapy. Patients are often required to cooperate for a long time and stay in a constrained position: this may affect their psychological state and induce discomfort, fear, anxiety, and pain ^1^.

The term Dental Anxiety (DA) includes anxiety, fear and phobia which are used interchangeably ^2^. DA is a reaction to unknown perceived dental danger especially when the treatment proposed was never experienced before. Dental fear is a reaction to a known perceived danger which involves a flight-or-fight response when provoked with the frightening stimulus, while dental phobia is an extreme, marked, and persistent fear of clearly visible defined objects or situations.

Understanding the level of patient anxiety allows its appropriate management. However, anxiety is difficult to measure. There are several methods available for dentist to score patients dental anxiety for example the Modified Child Dental Anxiety Scale (MCDAS) ^3^.

When patients can arduously cooperate during treatments, an appropriate analgesia should be achieved and a satisfactory anxiolysis should be accomplished. To this aim, a range of techniques, from the tell-show-do approach to conscious sedation and general anaesthesia, is currently available ^4, 5^.

Conscious sedation is a drug-induced depression of consciousness where the patient purposefully responds to verbal commands, either alone or by light tactile stimulation. No interventions are required to maintain a patent airway, and spontaneous ventilation is adequate. Patient’s cardiovascular function is usually maintained ^6^.

Meticulous pre-sedation evaluation with respect to patient’s general health status, airway, fasting, and understanding about the pharmacodynamics and pharmacokinetics of the drugs must be recognized. Availability of airway management equipment, sedative drugs’ antidote, venous access, suitable intraoperative monitoring such as pulse oximetry and well-trained staff must be ensured ^7^.

Conscious sedation can be administered through various routes such as oral, intramuscular, intravenous, and inhalational.

When fearful patients cannot be reassured by explanation and comforting professional behaviour, the clinician should consider a well-tolerated and effective sedative agent, associated with the use of topical analgesia and/or local analgesia (LA) injection.

The plurality of strategies available enables the team to safely provide a comprehensive care to minimize patient discomfort. Treatments include the use of topical analgesia on the gingivae prior to LA injection, anxiolysis with oral benzodiazepines, conscious sedation with nitrous oxide, intravenous conscious sedation with benzodiazepines (in these cases lidocaine and/or prilocaine creams are applied on the skin as an aid for venipuncture), and general anaesthesia ^8– 12^.

These techniques should be tailored based on specific individual needs and safety, to minimize anxiety, pain and memory of the dental procedure. To date, in the scientific literature, there is a lack of evidence based procedures to ensure patient’s compliance, considering both safety and cost-to-benefit ratio.

In this case series, five clinical cases (2 men and 3 women) and the procedures adopted to manage pain, behaviour and discomfort are analysed. We applied a patient-centered model to maintain patient’s cooperation and manage patient’s response to distress according to his/her psychological profile and the planned dental therapies.

## Case 1: a patient with low stress, good compliance

A 38-year-old woman with spontaneous bleeding during the night and during dental hygiene, halitosis, and difficulty in hygiene maintenance, came to our private practice. She was diagnosed as suffering long-term periodontal disease. At the first visit, the patient was relaxed and confident with the environment and the dental team. After a comprehensive periodontal visit, the patient received all the information about her disease and oral hygiene maintenance, and two dental chair appointments for dental hygiene were scheduled. We discussed the available procedures for pain and distress control; however, she refused any medication as she exhibited very low stress and good compliance (evaluated by anamnesis, counselling and clinical observation). She only accepted the use of topical analgesia on the gums (Lidocaine 15% spray). Both cleaning procedures were performed without distress and the patient was successfully treated for a form of periodontitis. Afterwards, the patient was included in a six month-recall programme for dental hygiene. The patient reported in each follow-up visit satisfaction for the previous treatment.

For patients with low stress and good compliance, undergoing dental treatments or procedures, such as dental hygiene, X-ray examinations, impression taking, simple stages of prosthetic rehabilitation and restorative dentistry, dentist behaviour and the use of topical analgesia (paste or liquid) is sufficient to control pain and manage the patient.

## Case 2: a patient with moderate stress and reduced compliance

A 34-year-old woman was referred to our private practice by a psychologist: she had suffered panic attacks and agoraphobia (extreme or irrational fear of entering open or crowded places, of leaving one’s own home, or of being in places from which escape is difficult) and had been in psychological therapy for about five years. The patient needed professional dental hygiene and some restorations.

The treatment planning was exhaustively explained to the patient.

At the first visit, the patient revealed slightly uneasiness and was unsettled and tense: she disclosed her discomfort while being subjected to dental procedures and enduring dental chair appointments and she was worried about potential pain. After we had had a consultation with patient’s psychologist and after we had also explained to him the planned dental treatment, in agreement with him, we used topical analgesia prior to LA injection (Topical analgesia: Lidocaine 15% spray; LA injection: Mepicain 2%, 1.8 ml, 1:100.000 adrenaline, or Mepicain 3%, 1.8 ml, without adrenaline) and a benzodiazepine by oral route ( *per os)* (10 drops of bromazepam were administered, 30 minutes before each dental procedure). All dental procedures were performed within a reasonable length of time with the patient remaining comfortable throughout the dental chair appointments. The patient reported satisfaction to her psychologist for the received treatments and, thus, the same regimen of anxiolysis was maintained during the following dental chair appointments.

Restorative dentistry, endodontic therapies, scaling and root-planning therapies are sometimes well tolerated since they have a low intensity of physical discomfort and low grade of psychological effort. However, for certain patients, the use of *per os* benzodiazepines is advisable in addition to local analgesia.

## Case 3: an anxious patient

A 54-year-old male needed the extraction of an upper wisdom tooth; the patient complained of pain, posteriorly, on the upper left side of the maxilla. After clinical and radiographic examinations, the upper left wisdom tooth was found with deeply a carious lesion with pulpal involvement, causing the pain.

The patient seemed to be cooperative, compliant and confident with the dental team and the surgery, even though the clinical history and medical record showed previous episodes of moderate anxiety related to dental procedures. Before the surgical intervention, topical analgesia (Lidocaine 15% spray) was used prior to LA injection. LA injection was administered on the vestibular and palatal aspects, for both pain control and vasoconstriction (Ecocain, 2x 20 mg/ml, 1,8 ml, 1:50:000 adrenaline). However, due to pulpal involvement and the presence of an infection, good pain control was not achievable ^13^. The patient showed distinct and comprehensible signs and symptoms of distress and an inability to withstand that surgical situation and the surgical steps. Thus, he rapidly became nervous and agitated. Not to lose the confidence and trust of the patient, we administered a benzodiazepine (bromazepam 15 drops *per os*) and nitrous oxide (Inhalation; Start: 10% nitrous oxide and 90% oxygen, progressively reaching 40% nitrous oxide and 60% oxygen). After 20 minutes of relaxation, we could perform the extraction with the perfect compliance of the patient and a better control of the intraoperative pain. At the end of the procedure, we administered 100% oxygen for 10 minutes for patient recovery. The patient reported satisfaction for the previous treatment at the suture removal visit.

When facing a patient with special needs (anxiety and reduced compliance), especially before procedures such as a planned minor surgery, or an extraction or a mucogingival surgery or an osseous periodontal surgery or a simple case of implantology, the dental team should preserve patient confidence, reduce anxiety and obtain compliance. In addition to local analgesia, the dentist should be prepared to supplement LA infiltration with inhalation sedation and/or oral sedation, even in the absence of the specialist anaesthesiologist.

The combination of benzodiazepines and nitrous oxide positively affects both the patient and the dental professional: in fact, a good consciousness of time and space perception and deep conscious sedation are achievable by adjusting nitrous oxide titration; the dentist is more relaxed and concentrated on the procedure. Analgesia is then well controlled by means of local analgesia.

## Case 4: a patient with acute anxiety and emotional distress

A 58-year-old female in good general health status required some restorative dentistry and complex implant-prosthetic rehabilitations on both arches.

She suffered from acute anxiety and emotional distress; she repeatedly asked for explanations before and after each planned dental treatment. The dental treatment planned for the patient included sinus lift procedures on both sides of the maxilla and concomitant implant placement. Bone harvesting from the malar surface of both maxillary bones by a scraper was also planned. Therefore, the planned procedure was complex and was explained to the patient in simple language with empathy, taking into consideration her acute anxiety and emotional stress. For this reason and accounting for her emotional state, intravenous sedation was planned for the patient (Diazepam: starting dose 7mg; then 0.6 mg every 25 minutes were administered up to 20 minutes before the end of the procedure). A 2-hour observation period followed the surgery to allow the patient sufficient time for full recovery. Administering sedation and post-operative anti-inflammatory drugs intravenously was helpful. Consciousness of time and space perception was obtained, with complete recovery and full patient’s satisfaction. The patient maintained a good compliance and reported satisfaction during the next dental chair appointment. During the following appointments, to complete the prosthetic phases, the patient was managed by means of *per os* benzodiazepines (bromazepam 15 drops 30 minutes before each dental procedure) and topical analgesia prior to LA injection, when requested by the procedure (topical analgesia: Lidocaine 15% spray; injected local analgesia: Mepicain 2%, 1.8 ml, 1:100.000 adrenaline).

Intravenous sedation is an effective therapy for patients suffering acute anxiety and distress while undergoing dental procedures. It offers several advantages: the patient may become completely relaxed, depending on the deepness of sedation obtained by drug titration, compliant and with a good consciousness of time and space perception. In this condition, even major surgery can be accomplished, with a reduced duration of the dental procedure and lower stress for the dental practitioner. Intravenous sedation has been successfully administered in patients undergoing complex surgical procedures, such as bilateral sinus lifting and bone grafts and implants or bilateral vertical and lateral augmentation procedures with bone harvesting. Through the same vein utilised to administer conscious sedation drugs, it is advisable to administer also anti-inflammatory drugs for the immediate postoperative phase. The patient should be in good health and it is desirable to give the patient postoperative time for recovery and to have someone accompanying him/her back home. The dental office should have a good professional relationship with a specialist anaesthesiologist and higher costs should be expected for the patient.

## Case 5: an anguished patient

A 50-year-old male in excellent general health condition needed to undergo fixed implant-supported dental rehabilitation. Despite the strong motivation to be rehabilitated and the awareness of the related surgeries, at the first visit the patient was stressed about the dental therapies, exhibiting distress and anguish.

From an oral point of view, the patient was afflicted by severe periodontal disease. He had lost all the teeth of the left side of the maxilla, except the central incisor; bone resorption of the area was massive with the tongue interposing between the arches laterally. The periodontal disease was progressively well controlled by proper therapies and a bone grafting of the left side of the maxilla was planned. According to the literature, autogenous bone is considered the gold standard for bone grafting procedures ^14^; therefore, calvaria was selected as donor site, to harvest bone blocks to perform vertical and horizontal bone augmentation in the large area for proper dental implants insertion and stabilization. Sinus lifting was planned concomitantly with bone augmentation. In this case, the unique appropriate sedation technique was general anaesthesia (Propofol; Starting dose: 144 mg; then 640 mg per hour of continuous venous infusion up to 15 minutes before the end of the surgical procedure; then 320 mg per hour of continuous venous infusion up to 5 minutes before the end of the surgical procedure; then the infusion was stopped). Local analgesia injection was also administered in the mouth and in the parietal bone area of the patient, to obtain vasoconstriction (Ecocain, 6 × 20 mg/ml, 1.8 ml, 1:50:000 adrenaline). After 5 months, five endo-osseous dental implants were placed. During the previous appointments for dental care procedures, the dental team gradually gained patient’s confidence, so that the surgical stage of implant placement and the other prosthetic phases were performed with local analgesia (Topical analgesia: Lidocaine 15% spray; injected local analgesia: Mepicain 2%, 1.8 mm, 1:100.000 adrenaline) or, rarely, with benzodiazepines *per os* (bromazepam 15 drops, 30 minutes before each treatment). All the implants were prosthetically rehabilitated four months after implant placement.

General anaesthesia is of paramount importance for the anguished patient while undergoing dental treatment. It may also represent the only chance to treat children under 4 years of age or to accomplish particular surgical procedures, such as bone harvesting from hip or calvaria for successive implant stabilisation or interventions in the extreme proximity to vascular or nervous anatomical structures.

The patient should be in good general health, hospitalisation is essential for the patient as well as the presence of a specialist anaesthesiologist. Generally, it is advisable to guarantee at least 24 hours for full recovery. Costs may eventually increase.

## Discussion

This case series describes five scenarios that frequently occur in clinical practice, with the evident limit of showing only few of the main typology of distress and pain management during dental procedures. Facing a wide range of patients, from the relaxed and collaborative to the anguished ones, the dental team should optimize and tailor the approach, considering both patients’ psychological profiles and the planned procedures.

The applicability and, at the same time, the limit of the described approach is that the evaluation and treatment of anxiety and distress was made by clinical observation and counselling and patient self-report, and was not based upon a mental health specialist evaluation and treatment (questionnaire of rating scale, psychotherapy or psychiatric consultation), since they are not routinely available in clinical dental practice.

During the treatments, patients are required to cooperate to achieve a degree of relaxation sufficient to maintain the necessary constrained position. Therefore, in patients with a pre-existing psychiatric history, psychosocial maladjustment or psychological problems such as anxiety and previous traumatic experiences related to dental therapies, behavioural manifestations can hinder the correct delivery of dental therapies, jeopardizing safety and clinical outcomes ^15^.

Before choosing the type of behavioural-sedative-analgesic approach, preoperative diagnosis, patient psychological profile, and the planned dental procedure should be carefully evaluated. The final aim is to perform the therapies with relaxed and cooperating patients and in an uneventful and smooth induction of analgesia and sedation, if needed. If well-balanced, sedation can convert the behaviour of an uncooperative patient, getting him/her suitable to undergo dental treatment.

Our experience indicates that it is necessary to adopt the most appropriate combination of resources ( Table 1), according to patient’s characteristics and needs. Therefore, we propose a decision-making strategy for the dental clinician, as a tool to maintain an acceptable level of collaboration and, therefore, of patient comfort during dental procedures. Clinical management strategy, analgesic agent and sedation therapy should be driven by the subjective perception of the patient. Besides generally utilized medications for pain control, the dental clinician can discuss with the patient to determine the best approach to reduce distress and anxiety and properly manage the clinical scenario, considering all the available techniques of sedation.

**Table 1.** Basic resources for the patient-centered model.

Resource	
Behaviour	The patient should be reassured and entertained before and during the dental procedure intended to be accomplished. Explanations must be given before each treatment.
Local analgesia	Topical and/or injectable analgesics and, if needed, sedatives can be chosen to both achieve pain relief and gain patient’s compliance and cooperation. Failure to achieve complete local analgesia is not uncommon in the management of orofacial infections ^13^: a shift to a deeper sedation may be required.
Anxiolytics	Use of anxiolytic drugs may be indicated to manage some disorders that cannot be addressed only with psychological and relational tools but need psychopharmacological therapy; the use of low-dose benzodiazepines to reduce anxiety can be very helpful. Patients’ responses to medications and doses can vary dramatically (i.e. from a moderate sedation to unintended deep sedation).
Procedural sedation	A complex of techniques to manage patient’s pain and anxiety in a safe, effective and human fashion ^11^ by maintaining spontaneous breathing and airway-protective reflexes ^12^. The patient should be carefully evaluated and prepared before sedation and monitored during the procedure and recovery. Sedation could be titrated and reduced in future treatments, based on the patient’s response. This technique is specially indicated for those individuals who are terrified of dental treatment.
General anaesthesia	It requires an accurate evaluation of costs and benefits, the presence of good general conditions, hospitalisation, and the presence of an anaesthesiologist.

Therefore, in this patient-centered model, the clinical decision of the behavioural support and of the proper analgesia and/or sedation is guided by patient complexity.

Therefore we modulated and balanced our behaviour and/or analgesia and/or sedation in response to a rising level of anxiety and distress, as in clinical cases of increasing complexity and/or length of the procedure (e.g. in cases of adverse events) ( Table 2). Moreover, a patient with strong anxiety, even if undergoing a generally tolerable procedure, may still require highly complex anxiolytic and analgesic procedures (as in cases 3 and 4). Additionally, if a level of analgesia is not feasible (e.g. in cases of local analgesia failures due to the presence of an infection), the next level of behavioural approach and anxiolysis should be considered. As another example, when the maximum dosage of analgesia or sedation has been achieved for a specific patient, it is convenient to use all the available behavioural techniques and as much empathy as possible to complete complex procedures with difficult patients.

**Table 2.** 

Procedure	Behaviour	Analgesia	Sedation
**From degree 0 to +++**	+	+	+
	++	+	0
	++	0	+
	+	++	0
	0	++	+
	+	0	++
	0	+	++
	+++	0	0
	0	+++	0
	0	0	+++

0: no increment of behaviour, analgesia or sedation; +: small increment of behaviour, analgesia or sedation; ++: medium increment of behaviour, analgesia or sedation; +++: high increment of behaviour, analgesia or sedation.

Along specific goals of sedation, the safety and prompt recovery to a state of consciousness must be considered. Furthermore, dentist’s preparation, expertise and experience can decrease the duration of the procedure, thereby limiting the need for sedation and analgesia ^16, 17^.

Nevertheless, on the basis of our experience we learnt that it is still a subjective process and each clinician must always be aware of patient’s response to sedatives. The more complex the procedure and/or as the length of the procedure increases, the more the dentist’s behaviour and/or analgesia and/or sedation need to match the new situation. The patient-centered approach still needs more studies to validate operative models to choose behavioural, sedative and analgesic strategies to achieve the best patient satisfaction.

Most anxious patients can be managed by proper communication and utilizing different behaviour management techniques (e.g. Tell-Show-Do, Distraction, Modelling, etc.) tailored to the patient psychological state. The clinician should strive to perform dental treatment using proper communication, behaviour management and local analgesia with or without oral and inhalational conscious sedation. Intravenous sedation and general anaesthesia should be reserved to complex cases to avoid potential morbidities and mortalities and additional treatment costs.

In conclusion, a patient-centered approach considering both clinical characteristics and psychological profile can help to achieve high quality dental care through a tailored management of pain and anxiety in patients with emotional and behavioural problems.

This can be achieved by proper behavior management and local analgesia with or without conscious sedation or in complex cases using intravenous sedation or general anaesthesia.

## Consent

All patients gave their written informed consent to publish the data presented in this case series.

## Data availability

All data underlying the results are available as part of the article and no additional source data are required.
