# Supplementary material for: A case series analysing patients with dental anxiety: a patient-centered model based on psychological profiling
Source: F1000Res. 2020 Sep 4;8:1843. Originally published 2019 Nov 1. [Version 2] doi: 10.12688/f1000research.20712.2 (PMC7525336; doi:10.12688/f1000research.20712.2)
Supplement: Peer review history

# Peer review: Reviewer response for version 2

**DOI:** 10.5256/f1000research.28309.r70853

**Published:** 2020-09-29

**Authors:** Senem Yildirimturk

**Affiliations:** 1 Department of Oral and Maxillofacial Surgery, Istanbul University, Istanbul, Turkey

Thanks to the authors.

Are enough details provided of any physical examination and diagnostic tests, treatment given and outcomes?

No

Is the background of the cases’ history and progression described in sufficient detail?

No

Is sufficient discussion included of the importance of the findings and their relevance to future understanding of disease processes, diagnosis or treatment?

No

Is the conclusion balanced and justified on the basis of the findings?

Partly

Reviewer Expertise:

Oral surgery, sedation, dental fear, dental anxiety, oral radiology,  cbct, dental implantology, facial fractures, oral and maxillofacial surgery

I confirm that I have read this submission and believe that I have an appropriate level of expertise to confirm that it is of an acceptable scientific standard.

# Peer review: Reviewer response for version 2

**DOI:** 10.5256/f1000research.28309.r70854

**Published:** 2020-09-07

**Authors:** Mawlood Kowash, Juma Alkhabuli

**Affiliations:** 1 Hamdan Bin Mohammed College of Dental Medicine, Mohammed Bin Rashid University of Medicine & Health Sciences, Dubai, United Arab Emirates; 2 RAK Medical and Health Sciences University, Ras Al-Khaimah, United Arab Emirates

The authors have satisfactorily addressed the concerns we raised in our previous peer review report.  We have no further comments to make.

Are enough details provided of any physical examination and diagnostic tests, treatment given and outcomes?

Partly

Is the background of the cases’ history and progression described in sufficient detail?

Partly

Is sufficient discussion included of the importance of the findings and their relevance to future understanding of disease processes, diagnosis or treatment?

Partly

Is the conclusion balanced and justified on the basis of the findings?

Partly

Reviewer Expertise:

Dental management of patients with special needs and behavioural problems.

We confirm that we have read this submission and believe that we have an appropriate level of expertise to confirm that it is of an acceptable scientific standard.

# Response

**Published:** 2020-09-07

**Authors:** Riccardo Tizzoni

**Affiliations:** Private Practice, Italy

Dear Doctors Mawlood Kowash and Juma Alkhabuli we thank you very much for the entire, precious process of reviewing our article.

Thank you very much.

I send you my Best Regards

Riccardo Tizzoni

# Peer review: Reviewer response for version 1

**DOI:** 10.5256/f1000research.22779.r65973

**Published:** 2020-07-13

**Authors:** Senem Yildirimturk

**Affiliations:** 1 Department of Oral and Maxillofacial Surgery, Istanbul University, Istanbul, Turkey

Here are my comments to the authors of 'A case series analysing patients with dental anxiety: a patient-centred model based on psychological profiling [version 1; peer review: 1 approved with reservations]':

1. If this study is an original research/research article, then what is the null hypothesis of the study?  If it is a review, the authors should better enhance the discussion and strongly support it with literature conducting dental anxiety, MDAS, sedation, etc. The examples given in the study (one in each type) are not enough to make a review!
2. The introduction and discussion parts are too short.
3. The authors should better use valid scales or questionnaires to avoid subjectivity. The dental history of the patient mostly affects the current state/mood on the day or even before the day of dental appointment regardless of operation type.
4. The grammar is poor.
5. The aim and methodology of the study should be revised and planned in detail.

Are enough details provided of any physical examination and diagnostic tests, treatment given and outcomes?

No

Is the background of the cases’ history and progression described in sufficient detail?

No

Is sufficient discussion included of the importance of the findings and their relevance to future understanding of disease processes, diagnosis or treatment?

No

Is the conclusion balanced and justified on the basis of the findings?

Partly

Reviewer Expertise:

Oral surgery, sedation, dental fear, dental anxiety, oral radiology,  cbct, dental implantology, facial fractures, oral and maxillofacial surgery

I confirm that I have read this submission and believe that I have an appropriate level of expertise to state that I do not consider it to be of an acceptable scientific standard, for reasons outlined above.

# Peer review: Reviewer response for version 1

**DOI:** 10.5256/f1000research.22779.r65974

**Published:** 2020-07-07

**Authors:** Mawlood Kowash, Juma Alkhabuli

**Affiliations:** 1 Hamdan Bin Mohammed College of Dental Medicine, Mohammed Bin Rashid University of Medicine & Health Sciences, Dubai, United Arab Emirates; 2 RAK Medical and Health Sciences University, Ras Al-Khaimah, United Arab Emirates

The five-case series of adult patients with varying degrees of anxiety and dental treatment proposes a patient-centred model to optimize patients’ cooperation during dental care delivery. Dental treatment of the five patients was achieved with behaviour management and local analgesia alone or with oral and inhalation conscious sedation, intravenous sedation, or general anaesthesia.

 The reviewers suggest the following corrections to make the paper scientifically sound: English language revision, revising the introduction by adding: 1) a paragraph on definition and assessment of dental anxiety; 2) a paragraph on conscious sedation, patient’s assessment, and monitoring. The discussion may be improved by revising Table 2 and adding a paragraph to emphasize the importance of communication and utilizing the different behaviour management techniques to manage patient dental anxiety. Finally, revision of the conclusion is suggested.

**English Language Revision: **the whole paper needs English language editing. The authors should use the commonly used words/terms in academic writing.

  

- In the introduction: 1 ^st^ paragraph: change the word “for” to “from” either consultation or dental therapy. 3 ^rd^ paragraph: use of topical analgesia prior to Local analgesia (LA) injection- and not “venipuncture” which means intra-venous access.
- In Case 1, line 7: “House hygiene maintenance” to be changed in to “oral hygiene maintenance”.
- The authors used the term (analgesia) in the abstract which is the correct term for dental local analgesia (LA). However, they also used (anaesthesia) throughout the paper to describe dental LA. Analgesia means the absence of pain sensation, while anesthesia means loss of all sensation including pressure and proprioception. Therefore, we suggest changing the term “anaesthesia” with ‘analgesia” for dental LA.
- The abbreviations should be defined when first used in the paper:  e.g. Benzodiazepine per os:  is OS mean by oral route?
- Use the word “restorations” instead of “fillings”

 

- In case 2: First paragraph:  define “agoraphobia”:  extreme or irrational fear of entering open or crowded places, of leaving one's own home, or of being in places from which escape is difficult.
- In 2 ^nd^ paragraph, line 5 ”we decided to supply her…should be changed to ”The local analgesia is applied
- Case 3: In the last paragraph **:** change the word “association” to “combination” of benzodiazepine and nitrous oxide positively affects ….

 

- Case 5: line 8: change “reabsorption” to “resorption”.

 

**Revision of Cases**

**Case 1**

- Start with the patient complaint or the reason for attending (e.g. refereed by..)  and not by diagnosis.
- It is not clear how the stress level was evaluated or based on which criteria?

 

**Case 2**

- Consultation with the patient’s psychologist should have been done prior to subjecting the patient to complex dental treatment.
- The following statement needs revision because the mentioned dental procedures are anxiety and pain-provoking procedures and generally are not tolerated by anxious patients:

“Restorative dentistry, endodontic therapies, scaling and root planning therapies are generally well tolerated since they have a low intensity of physical discomfort and low grade of psychological effort. However, for certain patients, the use of per os benzodiazepine is advisable in addition to local anaesthesia.”

**Case 3**

- Needs language revision as shown:

A 54-year-old male needed the extraction of an upper wisdom tooth; the patient complained of pain posteriorly, to (on) the upper left side of the maxilla. After X-ray clinical and radiographic examinations, the upper left wisdom tooth was found diagnosed with deeply a carious lesion with pulpal involvement, causing the pain.

- As mentioned in case 2, for the patient presented in Case 3 proper treatment planning should have been carried out because “clinical history and medical record showed previous episodes of moderate anxiety related to dental procedures”.  In addition, he required extraction of pulpally involved upper third molar.  The infiltration LA should have been supplemented with inhalation sedation and/or benzocaine oral sedation.

 

- In the second paragraph, line 11: following sentence needs revision:

“immediately at the beginning of the procedure, the use of periosteals to break and cut gingival fibres and the use of forceps to move luxate the tooth for dislocation caused intense”

**Case 4**

- The following statement is inappropriate:

“She suffered from acute anxiety and emotional distress; she constantly asked for explanations before and after each planned dental treatment, with an extreme waste of time during dental chair appointments”.

 The procedures should have been explained to the patient in simple language with empathy, taking into consideration her acute anxiety and emotional stress. Treatment under IV sedation should have been planned because she was diagnosed with acute anxiety and emotional stress.

  

- A good “disturbance” to be changed to “consciousness” of time and space perception…”

 

**Introduction- **Add the following:

**Definition and assessment of dental anxiety**

 The term Dental Anxiety (DA) includes anxiety, fear and phobia which are used interchangeably (McDonnell-Boudra et al. 2014). DA is a reaction to unknown perceived dental danger especially when the treatment proposed was never experienced before. Dental fear is a reaction to a known perceived danger which involves a flight-or-fight response when provoked with the frightening stimulus, while dental phobia is an extreme, marked, and persistent fear of clearly visible defined objects or situations.

 Understanding the level of patient anxiety allows its appropriate management. However, anxiety is difficult to measure.  There are several methods available for dentist to score patients dental anxiety for example the *Modified Child Dental Anxiety Scale (MCDAS)* (Wong et al. 1998).

**Definition of conscious sedation, patient’s assessment, and monitoring**

 Conscious sedation is a drug-induced depression of consciousness where the patient purposefully responds to verbal commands, either alone or by light tactile stimulation. No interventions are required to maintain a patent airway, and spontaneous ventilation is adequate. Patient’s cardiovascular function is usually maintained (Galeotti et al. 2016).

 Meticulous pre-sedation evaluation with respect to patient’s general health status, airway, fasting, and understanding about the pharmacodynamics and pharmacokinetics of the drugs must be recognized. Availability of airway management equipment, sedative drugs’ antidote,  

 venous access, suitable intraoperative monitoring such as pulse oximetry and well-trained staff must be ensured (Attri et al. 2017).  Conscious sedation can be administered through various routes such as oral, intramuscular, intravenous, and inhalational.

  

- McDonnell-Boudra D, Martin A, Hussein I. In vivo exposure therapy for the treatment of an adult needle phobic. Dent Update. 2014;41(6):533–40.
- Wong HM, Humphris GM, Lee GTR. Preliminary validation and reliability of the modifed child dental anxiety scale. Psychol Rep. 1998; 83:1179–86.
- Galeotti A, Garret Bernardin A, D’Antò V, Ferrazzano GF, Gentile T, Viarani V, et al. Inhalation conscious sedation with nitrous oxide and oxygen as alternative to general anesthesia in precooperative, fearful, and disabled pediatric dental patients: A large survey on 688 working sessions. Biomed Res Int 2016. 2016:7289310. 
- Attri JP, Sharan R, Makkar V, Gupta KK, Khetarpal R, Kataria AP, et al. Conscious sedation: Emerging trends in pediatric dentistry. Anesth Essays Res. 2017; 11:277–81.

 

**Discussion**

- In table 2 the highlighted sentence needs deletion from the title.

**Table 2.** Modulating and balancing behaviour and/or analgesia and/or sedation in response to an increasing complexity and/or length of the procedure and increasing level of anxiety and distress. As an example, when it is not possible to use more analgesia or sedation, it is convenient to use all behavioural techniques to perform a complex procedure with a difficult patient.

- In Table 2: it is not possible to have a profound analgesia (+++) in a patient with (0 increment) behaviour and (0 increment) sedation.
- **Add the following at the end of the discussion:**

Most anxious patients can be managed by proper communication and utilizing different behaviour management techniques (e.g. Tell-Show-Do, Distraction, Modelling, etc.) tailored to the patient psychological status.The clinician should strive to perform dental treatment using proper communication, behaviour management and local analgesia with or without oral and inhalational conscious sedation.Intravenous sedation and general anaestheisa should be reserved to complex cases to avoid potential morbidities and mortalities and additional treatment costs.

**Conclusion: **May be improved as follows:

 In conclusion, a patient-centred approach considering both clinical characteristics and psychological profile can help to achieve high quality dental care through a tailored management of pain and anxiety in patients with emotional and behavioural problems. This can be achieved by proper behavior management and local analgesia with or without conscious sedation or in complex cases using intravenous sedation or general anaesthesia.

Are enough details provided of any physical examination and diagnostic tests, treatment given and outcomes?

Partly

Is the background of the cases’ history and progression described in sufficient detail?

Partly

Is sufficient discussion included of the importance of the findings and their relevance to future understanding of disease processes, diagnosis or treatment?

Partly

Is the conclusion balanced and justified on the basis of the findings?

Partly

Reviewer Expertise:

Dental management of patients with special needs and behavioural problems.

We confirm that we have read this submission and believe that we have an appropriate level of expertise to confirm that it is of an acceptable scientific standard, however we have significant reservations, as outlined above.

# Response

**Published:** 2020-07-23

**Authors:** Riccardo Tizzoni

**Affiliations:** Private Practice, Italy

Object: revisions to article **A case series analysing patients with dental anxiety: a patient-centred model based on psychological profiling**

Dear Reviewer,

Thank you for reviewing our manuscript. The comments and suggestions indicated by you have been precisely considered and we hope satisfactorily addressed.

Enclosed please find the revised version that incorporates the reviewers’ comments as follows:

REVIEWER:

1. The language of the whole article was revised.
2. We changed the word “for” with “from”.
3. We have explained the use of topical analgesia for the skin prior to venipuncture.
4. We specified the term “topical analgesia” prior to “local analgesia”.
5. We changed  “House hygiene maintenance” to  “oral hygiene maintenance”.
6. We changed the word, when appropriated, “anaesthesia”  with “analgesia”.
7. We specified the term “per os” (that means “by oral route”) when first used in the paper.
8. We changed the world “fillings” with term “restorations” as suggested.
9. We explainded the meaning of agoraphobia ( *extreme or irrational fear of entering open or crowded places, of leaving one’s own home, or of being in places from which escape is difficult)*
10. We modified the sentence “we decided to supply her” with “the local analgesia is applied”
11. We changed the word “association” with “combination” in case 3
12. We changed the word “reabsorption” with “resorption”

REVISION OF CASES

**Case 1**

- We started with the patient complaint or the reason for attending and not by diagnosis
- The stress level was evaluated by clinical observation and self report of the patient (and we added in Discussion this period: “ *The applicability and, at the same time, the limit of the described approach is that the evaluation and treatment of anxiety and distress had been made by clinical observation and counselling and patient self-report, but was not based upon a mental health specialist evaluation and treatment (questionnaire of rating scale, psychotherapy or psychiatric consultation), since they are not available in a routine clinical dental practice*)”.

 

**Case 2**

- The Consultation with the patient’s psychologist has been done prior to subjecting the patient to complex dental treatment.
- We slightly modified the meaning of the sentence as requested.

 

**Case 3**

- We revised the first paragraph as indicated.
- For point 2 of case 3, please see point 2 of Case 1.
- For the patient presented in Case 3 also we clarified proper treatment planning with phrase *“* *supplemental infiltration with inhalation sedation with nitrous oxide administration and / or oral sedation”.*
- In the second paragraph, line 11 we deleted the indicated phrase, and we emphasized patient inability to withstand the surgical situation and surgical steps.

**Case 4**

- We modified the phrase correctly indicated as inappropriate: 

- We changed to “consciousness” of time and space perception…”

We added:

1. a paragraph on definition and assessment of dental anxiety; 2) a paragraph on conscious sedation, patient’s assessment, and monitoring.
2. We revised Table 2 and added a paragraph to emphasize the importance of communication and utilizing the different behaviour management techniques to manage patient dental anxiety.
3. We added the suggested paragraph in discussion.
4. We revised the conclusions.

  

In table 2 we deleted, as requested, the highlighted sentence from the title and explained the same concepts in the text.

 

- We considered possible to add doses of local analgesia (+++) in patients in whom is not need an increment of behavioural effort and sedation.

 

We hope that our responses and revisions satisfy your request for revisions.

Best regards
